# Germ‐free and microbiota‐associated mice yield small intestinal epithelial organoids with equivalent and robust transcriptome/proteome expression phenotypes

**DOI:** 10.1111/cmi.13191

**Published:** 2020-03-04

**Authors:** Annika Hausmann, Giancarlo Russo, Jonas Grossmann, Mirjam Zünd, Gerald Schwank, Ruedi Aebersold, Yansheng Liu, Mikael E. Sellin, Wolf‐Dietrich Hardt

**Affiliations:** ^1^ Institute of Microbiology, Department of Biology ETH Zurich Zurich Switzerland; ^2^ Functional Genomics Center Zurich University of Zurich Zurich Switzerland; ^3^ Institute of Pharmacology and Toxicology University of Zurich Zurich Switzerland; ^4^ Institute of Systems Biology, Department of Biology ETH Zurich Zurich Switzerland; ^5^ Department of Pharmacology, Cancer Biology Institute Yale University School of Medicine West Haven Connecticut USA; ^6^ Science for Life Laboratory, Department of Medical Biochemistry and Microbiology Uppsala University Uppsala Sweden

**Keywords:** inflammasome, microbiota, organoids

## Abstract

Intestinal epithelial organoids established from gut tissue have become a widely used research tool. However, it remains unclear how environmental cues, divergent microbiota composition and other sources of variation before, during and after establishment confound organoid properties, and how these properties relate to the original tissue. While environmental influences cannot be easily addressed in human organoids, mice offer a controlled assay‐system. Here, we probed the effect of donor microbiota differences, previously identified as a confounding factor in murine in vivo studies, on organoids. We analysed the proteomes and transcriptomes of primary organoid cultures established from two colonised and one germ‐free mouse colony of C57BL/6J genetic background, and compared them to their tissue of origin and commonly used cell lines. While an imprint of microbiota‐exposure was observed on the proteome of epithelial samples, the long‐term global impact of donor microbiota on organoid expression patterns was negligible. Instead, stochastic culture‐to‐culture differences accounted for a moderate variability between independently established organoids. Integration of transcriptome and proteome datasets revealed an organoid‐typic expression signature comprising 14 transcripts and 10 proteins that distinguished organoids across all donors from murine epithelial cell lines and fibroblasts and closely mimicked expression patterns in the gut epithelium. This included the inflammasome components ASC, *Naip1‐6*, *Nlrc4* and *Caspase‐1*, which were highly expressed in all organoids compared to the reference cell line m‐IC_c12_ or mouse embryonic fibroblasts. Taken together, these results reveal that the donor microbiota has little effect on the organoid phenotype and suggest that organoids represent a more suitable culture model than immortalised cell lines, in particular for studies of intestinal epithelial inflammasomes.

## 1 INTRODUCTION

Epithelia constitute essential barriers that protect the inner organs of the body, facilitate uptake and secretion and coordinate immune responses (Allaire et al., 2018). Consequently, their biology has received significant attention. Due to the difficulty of keeping primary epithelial cells in culture, mechanistic studies of epithelial cell biology and physiology have traditionally relied on epithelial cell lines—transformed cultures established from carcinomas or produced by introducing oncogenes (Bens et al., 1996; Fogh & Trempe, 1975; Fogh, Wright, & Loveless, 1977; Scherer, Syverton, & Gey, 1953). Cell lines are easy to grow, can be maintained in culture indefinitely and allow flexible genetic and pharmacological manipulation. However, the transferability of results to the in vivo scenario is often limited (Antoni, Burckel, Josset, & Noel, 2015; Ben‐David et al., 2018; Niepel et al., 2019). This can be explained by poor mimicking of the complexity and interconnectedness inherent to epithelia in vivo (Antoni et al., 2015), the disruptive effects of cellular transformation and the gradual accumulation of genetic anomalies during prolonged culture (Ben‐David et al., 2018; Foulke‐Abel et al., 2014; Liu et al., 2019). Therefore, new, more stable and possibly more representative experimental models are needed.

Gut epithelial organoids offer an attractive alternative. Protocols for culturing and differentiation of primary blood‐derived cell types have existed for decades (Sallusto & Lanzavecchia, 1994; Stone & Takemoto, 1970). More recently, the cumulative knowledge of the signals that maintain stem cells, drive epithelial cell growth and promote differentiation has allowed analogous protocols to be developed for epithelia from humans and mice. This progress has been driven by studies of the gut stem cell niche (Sato et al., 2009; Sato & Clevers, 2013; Stappenbeck & Virgin, 2016). Embedding of extracted intestinal epithelial stem cells in a matrix overlaid with a growth factor‐enriched culture medium (containing, e.g., Wnt, Noggin, EGF, R‐spondin; Sato et al., 2011; Sato & Clevers, 2013) results in the outgrowth of three‐dimensional primary epithelial structures—so called intestinal epithelial organoids (hereafter simply referred to as “organoids”). These organoids comprise a single layer of epithelial cells, with their apical side oriented towards a central lumen, while the basal side faces the extracellular matrix. In further similarity to the intact gut, organoids feature crypt invaginations harbouring the stem cell compartment (Sato & Clevers, 2013). These stem cells divide and give rise to epithelial cell precursors, which differentiate into paneth cells, enteroendocrine cells, goblet cells and enterocytes, hence recapitulating much of the complexity of co‐existing cell types in the gut mucosa (Foulke‐Abel et al., 2014; Sato et al., 2011). For these reasons, organoids have since their conception become a widely used and realistic model to study the role of intestinal epithelial cells in, for example, gut physiology (Almeqdadi, Mana, Roper, & Yilmaz, 2019; Gunasekara et al., 2018; Williamson et al., 2018), cancer biology (Drost et al., 2015; Tuveson & Clevers, 2019), pharmacology (Takahashi, 2019; Walsh, Cook, Sanders, Arteaga, & Skala, 2016) and infectious disease (Co et al., 2019; Foulke‐Abel et al., 2014; Hausmann & Hardt, 2019; Sun, 2017; Zhang, Wu, Xia, & Sun, 2014). It appears conceivable that organoids over time will replace traditional cell lines as the main tissue culture model of choice for mechanistic studies. However, it has remained unclear if the donor gut environment, in particular microbiota exposure, affects the organoid phenotype. These factors are difficult to control in organoids derived from human donors. To address the influence of the donor microbiota on organoid cultures, we have here compared organoids from well‐controlled colonies of genetically identical mice, either germ‐free or colonised with two different microbiotas.

The role of previous microbiota exposure on organoid cultures is particularly interesting, as the gut epithelium is constantly exposed to signals from environmental substances and intestinal microbes (Allaire et al., 2018). The microbiota can profoundly impact diverse aspects of epithelial physiology, including autophagy, mucus production and antimicrobial defence mechanisms (Benjamin, Sumpter, Levine, & Hooper, 2013; Chen et al., 2018; Jakobsson et al., 2015). Certain microbiota members even influence intestinal epithelial stem cell numbers and their proliferative capacity in vivo (Lee et al., 2018; Pan et al., 2018; Reedy, Luo, Neish, & Jones, 2019; Savage, Siegel, Snellen, & Whitt, 1981; Sommer & Bäckhed, 2016; Stecher et al., 2006). As a result, the non‐equal microbiota composition between separately kept mouse lines represents a major confounding factor in studies of how host genetics affect gut physiology and disease (Hausmann & Hardt, 2019; Mamantopoulos et al., 2017; Mamantopoulos, Ronchi, McCoy, & Wullaert, 2018; Robertson et al., 2019; Stappenbeck & Virgin, 2016). Especially in the fields of gut inflammation and infection biology, the necessity for littermate‐controlled in vivo experiments to normalise for such microbiota effects has become pressingly evident (Mamantopoulos et al., 2018).

The stem cell‐containing crypts that make up the starting‐material for intestinal epithelial organoids derive directly from this complex in vivo niche (Sato & Clevers, 2013). This raises the question whether environmental/microbial stimuli within the donor animal impact the long‐term phenotype of established organoid cultures, for example, by epigenetic mechanisms (Foster & Medzhitov, 2009) and whether experiments in genetically modified murine organoids require wild‐type littermate‐derived control organoid cultures. Moreover, the organoid establishment procedure itself might impose bottlenecks and promote drifts between independently generated cultures that could affect the long‐term phenotype. Hence, the impact of in vivo environmental factors, the amplitude and causes of organoid culture variability and the possible implications for experimental reproducibility remain poorly understood. This complicates the interpretation and comparability of results obtained in this emerging tissue culture model.

To assess reproducibility, faithful recapitulation of responses to relevant biological stimuli and stability towards confounding factors, we generated multiple independent organoid cultures from intestinal epithelial crypts of genetically identical mice housed in two distinct specific pathogen‐free (SPF) facilities and one germ‐free (GF) facility. By combining proteomics and transcriptomics, we compared the global expression profiles of the organoid cultures among each other, to their tissue of origin, and to widely used epithelial cell line and fibroblast models. Strikingly, organoids established from germ‐free or colonised mice exhibited basal expression profiles that co‐cluster together, rather than forming separate subgroups. Instead, the modest variability in expression between organoid cultures could be traced to stochastic sources during establishment and in‐culture maintenance. Also, the specific expression program induced by a defined stimulus—low‐dose TNF—appeared similar between organoid cultures from germ‐free and colonised animals, but differed markedly from TNF‐induced changes in a transformed intestinal epithelial cell line. Finally, our work uncovered an organoid expression signature that highlights significant expression of inflammasome signalling components in the primary intestinal epithelium, which is not detectable in commonly used cell lines.

## 2 RESULTS

### 2.1 Proteome profiles of independently established organoid cultures reveal a limited impact of the donor's microbiota

A tissue culture model should ideally exhibit limited variability and recapitulate the properties of the corresponding in vivo tissue. We have focused on murine intestinal epithelial organoids, as these provide an easily accessible system which allows precise control for impacts of the microbiota and the genetic background of the host, in contrast to human material. Using this system, we assessed reproducibility from genetically identical animals with the same life history, reared in the presence or the absence of a microbiota. Proteins carry most cellular functions and are tightly associated to specific phenotypes (Aebersold & Mann, 2016). Thus, as a start, we used proteome profiling to systematically probe the main sources of variability among intestinal epithelial organoid cultures and to address the relatedness of organoids to the gut epithelium.

To assess the effects of different microbiota exposures, we chose C57BL/6J wild‐type mice which were bred in parallel for >2 years (>10 generations) in two separate SPF facilities featuring two different microbiotas (SR and SE), and one germ‐free facility (GF). Organoid cultures were established from the jejunum of three 8–12 weeks old cohoused male littermate mice from each facility. During organoid establishment, samples corresponding to whole intestinal tissue (distal jejunum; contains epithelium, lamina propria and submucosa) (Tissue) and the isolated epithelial fraction (Epithelium) were also collected (Figure S1, Supporting Information). To avoid batch‐to‐batch medium variation, all organoid cultures were maintained using commercially available reagents (see section 4) purchased in bulk. Organoid cultures were grown to purity, cryopreserved in liquid nitrogen, revived and grown to passage 5–8 before sample collection (Organoid, see section 4). This sample set allowed us to probe the relatedness between primary intestinal epithelial cells and the corresponding organoids, and to assess the source(s) of inter‐sample variability in the absence of genetic diversity. As reference samples, we employed an immortalised murine small intestinal epithelial cell line (m‐IC_c12_; Bens et al., 1996). Mouse embryonic fibroblasts (MEF; C57BL/6 mesodermal origin) were chosen as an outgroup representing primary cells from a different mouse organ. Using SWATH mass spectrometry (SWATH‐MS; Aebersold & Mann, 2016; Gillet et al., 2012; Liu et al., 2015, 2019; Williams et al., 2016), a proteomic data acquisition method that generates highly reproducible datasets between multiple samples, randomised sample processing and downstream analysis in OpenSWATH (Röst et al., 2014, 2016), we were able to reproducibly quantify 3,653 Swissprot murine proteins (i.e., 3,331 unique proteins matching to the transcriptomics data below) across the entire sample set. Analysis of technical SWATH‐MS replicates confirmed a minimal variability stemming from the proteomics procedure itself (average Pearson correlation between technical replicates: 0.999).

**Figure 1:**
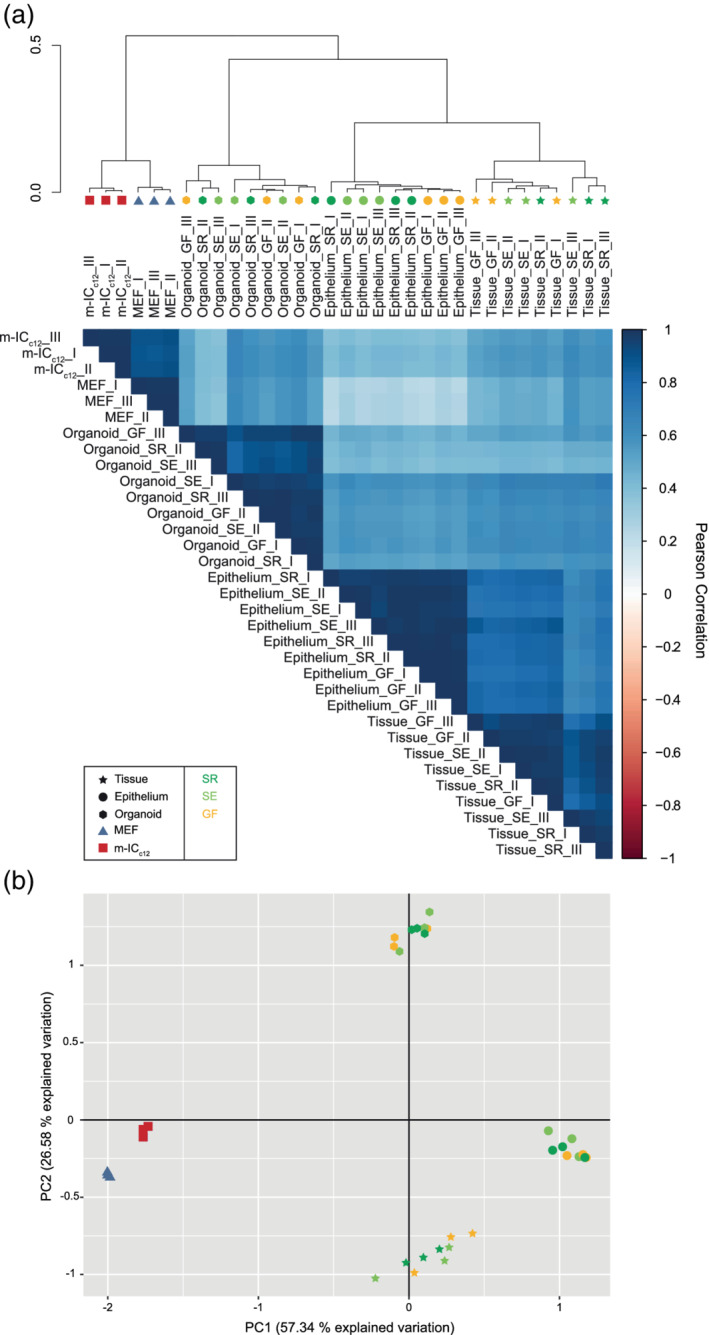
Donor microbiota minimally impacts the global protein expression pattern of small intestinal epithelial organoid cultures. (a) Unsupervised hierarchical clustering analysis of the proteome data set including tissue (Tissue_I‐III, star symbol), epithelial cell‐enriched fraction (Epithelium_I‐III, circle symbol) and organoid (Organoid_I‐III, hexagon symbol) samples from mice raised in SPF facility 1 (_SR, dark green), SPF facility 2 (_SE, light green) or the germ‐free facility (_GF, yellow), as well as MEF (MEF_I‐III, blue triangle symbol) and m‐IC_c12_ cell (m‐IC_c12__I‐III, red square symbol) samples. Correlation matrix depicts Pearson correlation values between indicated samples. (b) Principal component analysis of the proteome data set as described in (a)

Input from luminal microbiota, ingested chemicals and food particles may have profound effects on epithelial cell physiology and may imprint long‐lasting characteristics, for example, by epigenetic processes (Allaire et al., 2018; Lotz et al., 2006; Pan et al., 2018). We addressed whether such environmental conditions at the site‐of‐origin affected global proteome profiles within the sample set. In the unsupervised clustering, all three epithelium samples from the germ‐free facility (Epithelium_GF_I‐III) co‐clustered in a separate subgroup from the epithelium samples of the six SPF facility mice (Epithelium_SR_I‐III, _SE_I‐III) (Figure 1a). While the distances were small, these results are consistent with some impact of microbiota and/or other environmental stimuli on global epithelial cell protein expression within the gut. In contrast, the corresponding organoid samples (Organoid_GF_I‐III, _SR_I‐III and _SE_I‐III) were found to cluster essentially at random among each other (Figure 1a). Hence, source(s) of variation during establishment, cryopreservation, thawing, or continuous passaging appear to overshadow any impact of in vivo environment memory on the quantitative organoid proteome profiles.

Furthermore, the unsupervised clustering of the entire SWATH‐MS data set revealed that the organoid samples clustered together with the epithelium (average Pearson correlation: .527) and gut tissue sample groups (average Pearson correlation .595) (Figure 1a). The m‐IC_c12_ epithelial cell line sample group clustered further away in the dendrogram, and in fact was placed closer to the cultured MEFs than to either the organoid or epithelium sample groups (Figure 1a). Unsupervised clustering based on the top 100 proteins (as ranked by variance across all samples) instead of all proteins resulted in essentially identical results (Figure S2A). Moreover, a principal component analysis of the proteomes placed the organoid, the epithelium cell preparation (enriched for epithelial cells, see section 4 for details) and tissue samples in proximity to each other along the main principal component (PC) 1 axis of variation, with the m‐IC_c12_ and MEF samples at the opposing end of the axis (PC1 accounting for 57.34% of the variation, Figures 1b and S2B). The PC2 axis clearly resolved the organoid group from both epithelium and tissue (PC2 explaining 26.58% of the variation, Figures 1b and S2B). Hence, we conclude that stable small intestinal epithelial organoid cultures exhibit a distinct proteome profile, which shows appreciable similarity to the gut epithelium in vivo, and is largely unaffected by the gut microbiota of the tissue donor.

To gauge the level of experimental noise, we next assessed the variability in protein expression between replicates within each sample group (measured as dispersion coefficient; i.e., standard deviation divided by the mean, in percent). As expected, the two reference cell lines (m‐IC_c12_ and MEF) displayed a low variability between replicate samples (disp. coeff. of 13.27% and 11.77%, respectively; Table 1). A somewhat higher variability was noted across biological replicates within the tissue (disp. coeff. 19.73%) and epithelium (disp. coeff. 19.70%) sample groups. By comparison, the variability within the organoid sample group was lower than within the epithelial and tissue sample groups (disp. coeff. 16.12%; Table 1). Two of the organoid subgroups even displayed a variability close to the one of the m‐IC_c12_ sample group (disp. coeff. Organoid_SR 13.58%; Organoid_SE 17.08%; Organoid_GF 14.93%; Tables 1 and 2). Considering that each organoid sample stems from a unique establishment, cryopreservation, revival and ~3–4 additional weeks in separate culture, this degree of variability can be considered modest, and close to the variability noted for homogenous cell line cultures (m‐IC_c12_ and MEF). Moreover, the variability within the tissue and epithelium samples is higher than within the organoid sample group, indicating that environmental cues influencing mouse‐to‐mouse variations may be partially eliminated in culture.

**Table 1:** Dispersion coefficient of the different sample groups

Group	Dispersion coefficient (%)
Organoid	16.12
Tissue	19.73
Epithelium	19.70
m‐IC_c12_	13.27
MEF	11.77

**Table 2:** Dispersion coefficient of the different organoid groups

Group	Dispersion coefficient (%)
Organoid_SR	13.58
Organoid_SE	17.08
Organoid_GF	14.93

Taken together, we conclude (a) that murine small intestinal epithelial organoids exhibit a distinct proteome profile; (b) which resembles that of the in vivo epithelium more closely than an immortalised epithelial cell line; (c) that in vivo environmental factors including previous exposure to microbiota in the murine gut have a negligible impact on the global proteome of organoids; and (d) that the inter‐sample variability between independent organoid cultures is only modestly higher than for commonly used cell lines.

### 2.2 Contrasting stochastic organoid culture variation to the impact of a physiological stimulus

In a next step, we sought to contrast the stochastic variation between independently established organoid cultures to the impact of a subtle physiological stimulus. For this purpose, we stimulated organoids with the cytokine tumour necrosis factor (TNF) (5 ng/mL, 8 hr), known to induce a defined gene expression program in epithelial cells. Notably, we chose a low TNF concentration which would induce a distinct pro‐inflammatory response rather than cell death (Janes et al., 2006; Vlantis et al., 2016). This treatment led to the significant up‐ or down‐regulation of 15 proteins in the organoid sample group, including upregulation of typical marker proteins such as Nfkb2 (Mukherjee et al., 2017; Figure S3A and Table S1).

**Figure 2:**
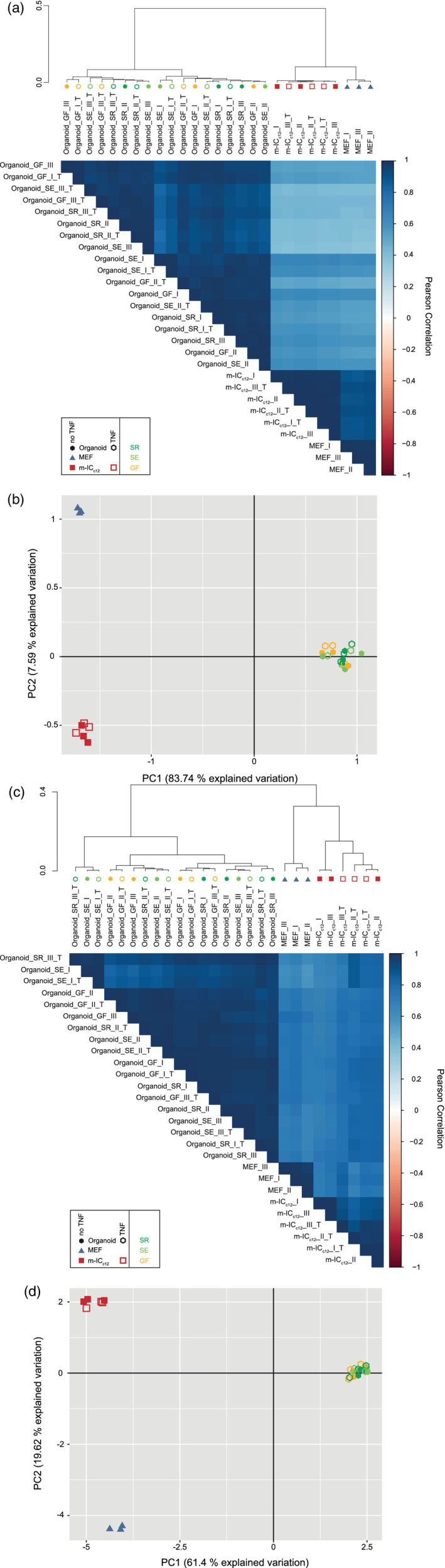
Stochastic variability between separate organoid cultures has a stronger impact on global gene expression than a defined, physiological stimulus. (a) Unsupervised hierarchical clustering analysis of the proteome data set including untreated (closed symbols) and TNF‐treated (5 ng/mL, 8 hr; open symbols;_T) organoid (Organoid_I‐III, hexagon symbol) samples from mice raised in SPF facility 1 (_SR, dark green), SPF facility 2 (_SE, light green) or the germ‐free facility (_GF, yellow), as well as MEF (MEF_I‐III, blue triangle symbol) and m‐IC_c12_ cell (m‐IC_c12__I‐III, red square symbol) samples. Correlation matrix depicts Pearson correlation values between indicated samples. (b) Principal component analysis of the proteome data set as described in (a). (c) Unsupervised hierarchical clustering analysis of the transcriptome data set for the samples described in (a). (d) Principal component analysis of the transcriptome data set as described in (c)

Again, the global unsupervised clustering was used to assess the relative impact of stochastic culture‐to‐culture variability and TNF‐induced changes. Notably, TNF‐treated organoid samples (_T) clustered among the untreated samples instead of forming a separate subgroup in the dendrogram (Figure 2a). A principal component analysis similarly revealed that all 18 organoid samples, irrespective of TNF‐treatment, formed one mixed group placed at the opposing end of the PC1 axis from the m‐IC_c12_ and MEF reference samples (Figure 2b). These data suggest a larger impact of stochastic or sample‐to‐sample variability (as shown in Figure 1a,b), than of subtle TNF stimulation upon the entire organoid proteome (Figure 2a,b).

To complement the proteome data, an identical analysis was also conducted at the transcriptomic level by Illumina HiSeq 4,000 sequencing (Figure 2c,d), which has a higher sensitivity for low‐abundance targets. We detected and mapped on average 4.26 × 10^7^ reads per sample. TNF treatment induced significant up‐ or down‐regulation of 316 out of 15,698 total transcripts (Figure S3b and Table S2). Among these are previously described TNF‐target genes, including *Nfkb2*, *Tnfaip3*, *C3* and *Relb* (Mukherjee et al., 2017; Sheerin, Zhou, Adler, & Sacks, 1997; Vlantis et al., 2016; Zhao et al., 2015). Again, neither unsupervised clustering nor a principle component analysis of the whole transcriptomes resolved the TNF‐treated organoid samples from the untreated sample group (Figure 2c,d). In fact, five out of nine TNF‐treated samples clustered closest to their non‐treated counterparts (see, e.g., Organoid_SE_I and Organoid_SE_I_T; Figure 2c). This is well in line with the subtle, physiological nature of the TNF stimulus employed in our experiment, in analogy to a typical specific biochemical perturbation, which is expected to affect only a very small set of selected genes in epithelial cells (Janes et al., 2006; Vlantis et al., 2016). Similar conclusions could be drawn both at the proteome and transcriptome level when the analysis was redone for the 100 proteins/transcripts contributing most to variation (Figure S4a–d). Hence, stochastic variability between separate organoid cultures has a stronger impact on the global expression pattern than the defined alteration of 15/3331 (0.45%) proteins and 316/15698 (2.01%) transcripts through low‐level TNF stimulation.

### 2.3 Robust induction of a TNF‐induced gene expression program in organoids from differentially colonised mice

**Figure 3:**
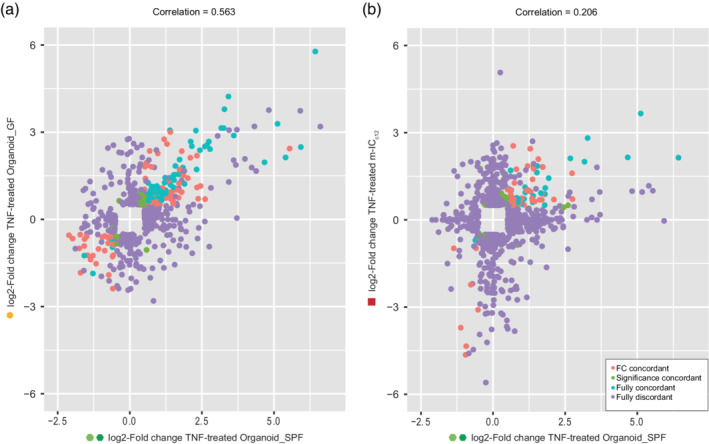
The TNF‐induced transcriptional response of organoids from germ‐free and colonised mice shows robust correlation. (a) Correlation plot of TNF‐induced changes in germ‐free mouse derived organoids (*y*‐axis, Organoid_GF) plotted against TNF‐induced changes in SPF‐derived organoids (x‐axis, Organoid_SPF). (b) Correlation plot of TNF‐induced changes in m‐IC_c12_ cells (*y*‐axis, m‐IC_c12_) plotted against TNF‐induced changes in SPF‐derived organoids (*x*‐axis, Organoid_SPF). Shown are fully concordant hits (blue), hits concordant in fold change (FC; red), hits concordant in *p*‐value (green) and fully disconcordant hits (purple). Cut‐offs: genes with log2 ratio <−0.5 or >0.5 and *p*‐value <0.05 and above background noise in at least one of the sample groups

The data above reveal a modestly elevated variability in baseline organoid gene expression, as compared to cultured cell lines (Figures 1 and 2). For an experimental model system to be useful, another key aspect is the ability to respond reproducibly to a given stimulus. To assess this, we estimated the similarity in specific gene expression changes induced by TNF stimulation, comparing the panel of transcripts significantly altered by TNF across organoids of different origin, that is, from the germ‐free facility (mean of Organoid_GF) and the two SPF facilities (means of Organoid_SR and Organoid_SE; “Organoid_SPF”). This analysis revealed that the organoids derived from germ‐free mice responded to TNF with a robust degree of similarity to those derived from SPF mice (*R* ^2^ = .563; Figure 3a). This implies that neither prior in vivo stem cell exposure/non‐exposure to gut microbes, nor variability in the organoid production process, imprint differences that may preclude interpretation of the small intestinal organoid responses to the prototypical stimulus TNF.

Traditionally, cultured cell lines have been used as a proxy for studies of cell signalling and gene expression regulation in the gut epithelium. We next compared the specific gene expression changes induced by TNF stimulation in m‐IC_c12_ cells versus SPF organoids. The correlation between significantly regulated transcripts was here considerably lower (Figure 3b, *R* ^2^ = 0.206). In fact, the vast majority of transcripts significantly up or down‐regulated in either the m‐IC_c12_ or the organoid group failed to show a corresponding behaviour in the other group (Figure 3b). We conclude that the gene expression changes induced by a physiological stimulus may vary substantially between an intestinal epithelial cell line and primary epithelial organoids from the same species. The variability in response between organoids from germ‐free and SPF mice, by contrast, appears more modest.

### 2.4 A small set of transcripts and proteins define an organoid‐typic expression signature

When applying unsupervised clustering to proteomes and transcriptomes of only the cultured cell sample groups (i.e., Organoids, m‐IC_c12_ cells and MEFs), we again found that in both cases, PC1 (explaining 83.56%/61.27% of the variation) clearly distinguished the organoid group from the other samples (S5A‐B). PC2 (explaining 8.2%/19.13% of the variation) defined a smaller variance distance between MEFs and m‐IC_c12_ sample groups (S5A‐B). This allowed investigation of the transcripts and proteins which define an organoid‐typic expression signature.

**Figure 4:**
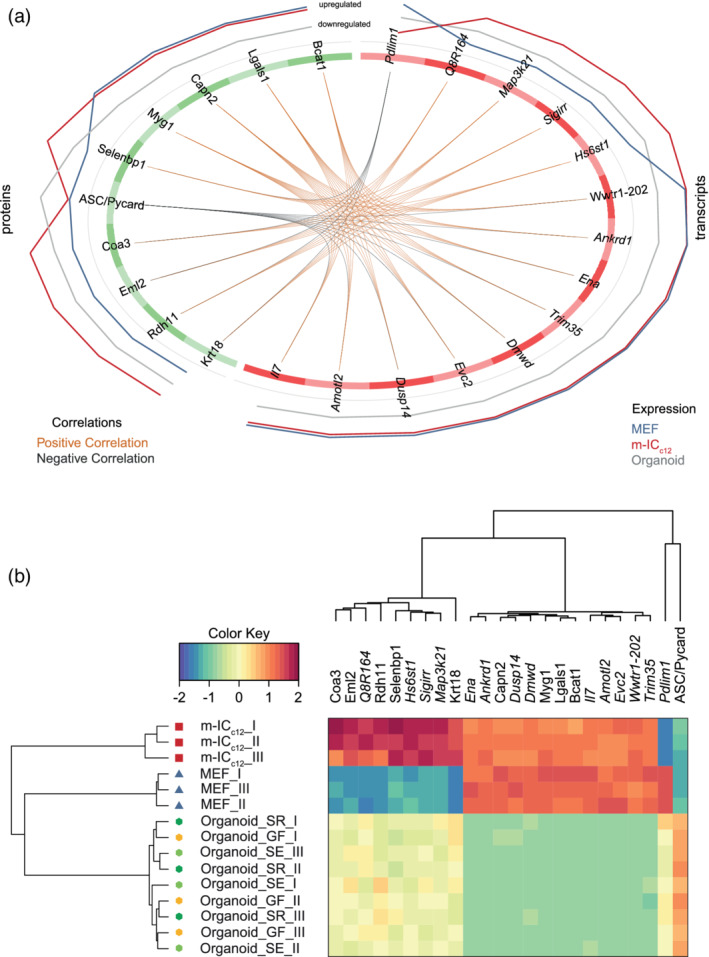
Identification of an organoid‐typic expression signature by integrative analysis. (a) Circos plot depicting the correlation of expression levels of the identified transcripts and proteins within the three analysed sample groups (Organoid (grey line), MEF (blue line) and m‐IC_c12_ (red line)). (b) Unsupervised hierarchical clustering analysis and expression heat map of the identified transcripts and proteins shown in (a) for Organoid (Organoid_I‐III, hexagon symbol) samples from mice raised in SPF facility 1 (_SR, dark green), SPF facility 2 (_SE, light green) or the germ‐free facility (_GF, yellow), as well as MEFs (MEF_I‐III, blue triangle symbol) and m‐IC_c12_ cell (m‐IC_c12__I‐III, red square symbol) samples

As a starting point, we performed an integrative analysis of the transcriptome and proteome data sets, using the DIABLO framework of the CRAN package mixOmics (Rohart, Gautier, Singh, & Lê Cao, 2017). The correlation between up‐ or downregulated proteins and their corresponding transcripts was high when comparing the organoid group to the MEF and m‐IC_c12_ groups (*R* ^2^ = .719) (Figure S5c). We identified 14 transcripts and 10 proteins that distinguished an organoid expression signature. Herein, the expression levels of nine transcripts (*Ena*, *Ankrd1*, *Dusp14*, *Dmwd*, *Il7*, *Amotl2*, *Evc2*, *Wwtr1‐202* and *Trim35*) and five proteins (Capn2, Myg1, Lgals1, Bcat1 and ASC/Pycard) contributed to PC1. Expression levels of five transcripts (*Q8R164*, *Hs6st1*, *Sigirr*, *Map3k21* and *Pdlim1*) and five proteins (Coa3, Eml2, Rdh11, Selenbp1 and Krt18) defined PC2 (Figures S5d, 4a,b, Table S3). Generally, expression levels of most transcripts and proteins within a PC positively correlated with each other (Figure 4a). The variables associated with the main PC1 were upregulated in the MEF and m‐IC_c12_ groups compared to the organoids. Here, ASC provided a notable exception, with substantially higher protein levels in organoids than in both MEFs and m‐IC_c12_ cells (Figure 4b). The transcripts/proteins that defined PC2 showed moderate expression in organoids, upregulated levels in m‐IC_c12_ cells and low expression in MEFs. Only the *Pdlim1* transcript behaved inversely between the MEF and m‐IC_c12_ groups (Figure 4b).

To test if the identified organoid signature agreed with expression levels in the gut epithelium, we reassessed expression of the 10 protein hits (Figure 4) in the entire proteome data set, that is, including also the tissue and epithelium sample groups (Figure S6). Strikingly, these identifier proteins showed highly similar expression levels in the epithelium samples and in organoids (Figure S6). Expression levels in whole intestinal tissue, which contains a mix of epithelial cells and multiple other cell types, appeared less similar. Finally, MEFs and m‐IC_c12_ samples formed a separate outgroup also in this comparison (Figure S6). Hence, we have uncovered a small set of transcripts and proteins that constitute a physiologically relevant small intestinal epithelial organoid signature.

**Figure 5:**
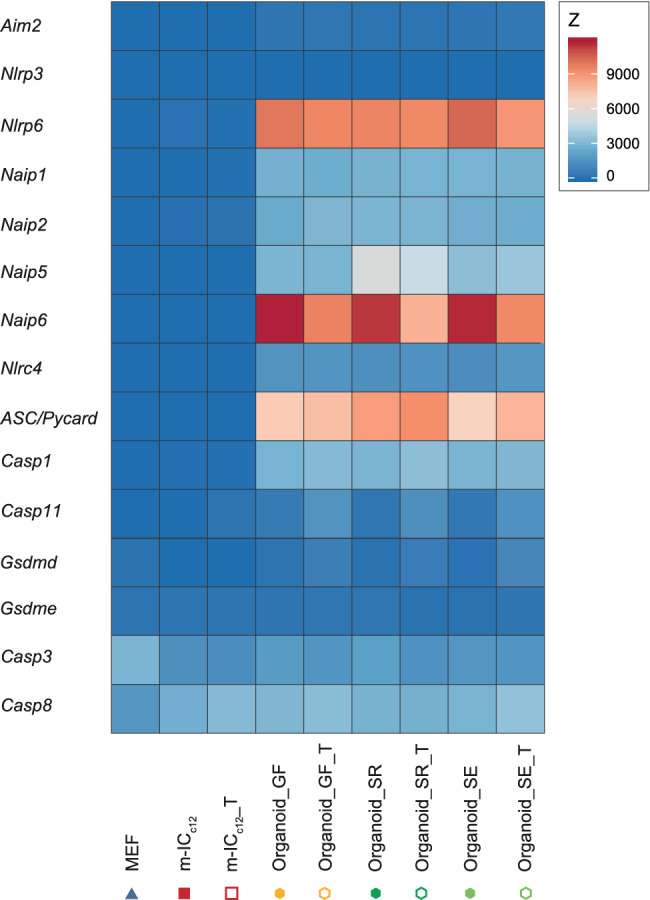
Inflammasome components are highly expressed in organoids compared to m‐IC_c12_ cells and fibroblasts. Heat map depicting expression levels of several inflammasome components in untreated or TNF‐treated (_T) organoids (Organoid_I‐III, hexagon symbol) samples from mice raised in SPF facility 1 (_SR, dark green), SPF facility 2 (_SE, light green) or the germ‐free facility (_GF, yellow), as well as untreated or TNF‐treated (_T) m‐IC_c12_ cell (m‐IC_c12__I‐III, red square symbol) and MEF (MEF_I‐III, blue triangle symbol) samples

Interestingly, the signature included high expression of the inflammasome‐scaffold protein ASC (apoptotic speck‐like protein; Figure 4b; Richards et al., 2001). Inflammasomes mediate responses to pathogen‐ (PAMP) or damage‐associated patterns (DAMP) by activation of Caspase‐1 or Caspase‐11, resulting in the release of active IL18 and/or IL1β, as well as induction of cell death (Broz, 2019). To probe whether additional inflammasome signalling components were also highly expressed specifically in organoids, we revisited the transcriptome data set. Strikingly, m‐IC_c12_ cells and MEFs showed low or undetectable expression levels for all inflammasome components analysed here (Figure 5). By contrast, organoids specifically expressed high levels of the NAIP/NLRC4 inflammasome components *Naip1*, *2*, *5*, *6* and *Nlrc4*, as well as *Asc*, *Nlrp6* and *Caspase‐1*. The expression of *Caspase‐11* and *Gsdmd* could be induced by TNF in organoids, whereas *Gsdme* and *Nlrp3* transcripts were not detectably expressed in any of the analysed sample groups. Finally, the pro‐apoptotic proteases *Caspase‐3* and *‐8* were expressed at similar levels across organoids and m‐IC_c12_ cells.

Taken together, these data suggest that intestinal epithelial organoids from differentially colonised donors exhibit a shared expression signature, which encompasses significant expression of inflammasome signalling components.

## 3 DISCUSSION

Our work validates that murine small intestinal organoids are an experimental system with a robust expression pattern that resembles the phenotype of the homeostatic intestinal epithelium (Janeckova et al., 2019; Lindeboom et al., 2018). Importantly, our work extends previous data by demonstrating that this phenotype is largely independent from exposure with SPF microbiota. This is in contrast to in vivo studies, in which microbiota divergence between separately held mouse lines can affect a range of physiological functions and lead to non‐reproducible results (Mamantopoulos et al., 2017, 2018; Robertson et al., 2019; Stappenbeck & Virgin, 2016). Even extensive co‐housing appears insufficient to fully homogenise the gut microbiota and its impact between previously separated animals (Robertson et al., 2019). Donor‐derived microbial cues in organoids grown in culture for ≥5 passages do not globally overshadow other sources of experimental variability. In line with this, organoids from SPF and GF donor mice showed a robust degree of concordance with respect to transcripts induced by low concentrations of the cytokine TNF. Littermate controls (which share the same microbiota) are a prerequisite for the accurate interpretation of in vivo gut biology data from knockout or transgenic animals. However, we here found that organoid experimentation may not require such littermate controls, as organoids from mouse colonies with or without microbiota yielded equivalent expression phenotypes. Notably, this applies to organoids cultured for ≥5 passages. It is likely that microbiota imprints are detectable in earlier passages (Janeckova et al., 2019).

The observed culture‐to‐culture variation in expression profiles may stem from bottleneck effects during early organoid establishment and/or adaptation to the culture conditions. In addition, the differentiation state and cell type composition of organoids is highly sensitive to the concentration of growth factors provided in the culture medium (e.g., Noggin, R‐spondin and EGF), or produced by the organoids themselves (e.g., Wnt3a; Farin et al., 2016; Kim et al., 2014; Lehmann et al., 2019; Lindeboom et al., 2018; Sato et al., 2009; van der Flier & Clevers, 2009). Fluctuation of these, often unstable, proteinaceous factors provides another plausible source of culture‐to‐culture variability. While the exact impact of these and potentially other causes remain to be examined, the net effect is a moderately higher variation in expression (~1.5‐fold increased dispersion coefficient for the proteome) among organoid cultures compared to the herein used reference cell lines. Considering that organoids more accurately mimic the overall expression patterns of the gut epithelium (this study), faithfully recapitulate the epithelial cell type composition of the intact gut, and in contrast to cell lines retain genetic stability over time (Ben‐David et al., 2018; Liu et al., 2019; Sato et al., 2009; Sato & Clevers, 2013), organoids nevertheless appear vastly superior to classical cell line models for predicting physiological responses in the intestinal epithelium.

Combined, our findings have implications for experimentation in intestinal epithelial organoids. As mentioned above, littermate control organoids appear oblivious, as microbiota differences between tissue donors do not confound the analysed global organoid phenotypes. Instead, the moderately elevated culture‐to‐culture variability may warrant larger experimental sample sizes overall to reliably detect subtle phenotypes. Finally, key findings should be validated in independently established organoid cultures from additional tissue donors to ensure reproducibility of results. It should be noted that these conclusions are strictly valid only for the small intestinal epithelial organoids of inbred specified pathogen free mice, as analysed in this study. We cannot exclude that persisting microbiota effects would be more pronounced in colon organoids, due to higher microbial exposure within this gut region in vivo. Nor do our data refute that some specific organoid signalling pathways can be affected by the tissue donor's microbial status, especially during early culture passages (Janeckova et al., 2019) or in cases of pathobiont exposure. Human inter‐individual variation also exceeds that of genetically inbred animals, which has repercussions for experimental design in patient‐derived organoids (Cristobal et al., 2017).

By integration of the transcriptome and the proteome datasets, we were able to identify a physiologically relevant organoid‐typic expression signature, distinguishing the full set of organoid cultures across all three SPF/GF mouse colonies from the reference cell lines. Interestingly, this signature highlighted high expression of ASC, a central scaffolding protein for inflammasome signalling pathways (Richards et al., 2001). Our follow‐up analysis extended this finding to also encompass the transcripts for a range of other inflammasome receptors (e.g., *Naip1*, *2*, *5*, *6*, *Nlrc4*), inflammatory caspases (e.g., *Caspase‐1*) and downstream executors (e.g., *GsdmD*), which all exhibited high expression in organoids and low to undetectable expression in epithelial cell line m‐IC_c12_ and fibroblast reference cells. Importantly, high expression of these inflammasome components in epithelial cells were reported previously (Hausmann, Sellin, & Hardt, 2020; Winsor, Krustev, Bruce, Philpott, & Girardin, 2019), further indicating that organoids more realistically represent the in vivo situation. The differential regulation of inflammasome components upon exposure to the proinflammatory cytokine TNF likely represents a preparation of epithelial cells to microbial exposure. Upon sensing of PAMPs or DAMPs, inflammasomes drive acute pro‐inflammatory and anti‐microbial responses (Broz & Dixit, 2016). However, earlier studies have also implicated, for example, ASC, NAIPs, NLRP3 and NLRP6 as tumour suppressors (Allam et al., 2015; Allen et al., 2010; Das et al., 2006; Normand et al., 2011). A hallmark feature of inflammasome activation is the prompt induction of cell death machinery in the activated cell (Aglietti et al., 2016; Kayagaki et al., 2011; Knodler et al., 2010, 2014; Miao et al., 2010; Rauch et al., 2017; Richards et al., 2001; Sellin et al., 2014; Shi et al., 2015). It therefore seems conceivable that upon transformation/immortalization of epithelial cell lines, there would be a strong selective pressure to lose or downregulate inflammasome pathway components and thereby dampen cell death effects. By contrast, organoids grown under optimal conditions retain expression also of such potential tumour suppressor genes. Notably, with regard to the widely discussed reciprocal interactions between microbiota and inflammasomes in the gut (Mamantopoulos et al., 2017; Robertson et al., 2019; Seo et al., 2015; Winsor et al., 2019), the expression of inflammasome components appears unaffected by the donor microbiota in small intestinal epithelial organoids. Thus, compared to classical tissue culture cell lines, organoids should be more realistic models to study the function of epithelial inflammasomes.

While the impact and mechanisms of inflammasome signalling in typical immune cells (e.g., macrophages, dendritic cells) have been thoroughly documented (Boyden & Dietrich, 2006; Franchi et al., 2006; Mariathasan et al., 2004; Martinon, Pétrilli, Mayor, Tardivel, & Tschopp, 2006; Miao et al., 2006), the importance of intestinal epithelial inflammasomes in tissue homeostasis and defence has become evident only recently (Allam et al., 2015; Harrison et al., 2015; Knodler et al., 2010, 2014; Nowarski et al., 2015; Rauch et al., 2017; Sellin et al., 2014; Winsor et al., 2019). Tumour‐derived or immortalised cell lines have traditionally been used as proxies for molecular studies in intestinal epithelia, which may in part explain why intestinal epithelial inflammasomes have for long been overlooked. We anticipate that the transition into primary organoids as the tissue culture models of choice will reshape our understanding of these and other physiological signalling circuits in the gut mucosa and beyond.

## 4 DATA ANALYSIS

### 4.1 RNA sequencing

Reads were quality‐checked with FastQC. Sequencing adapters were removed with Trimmomatic (Bolger, Lohse, & Usadel, 2014) and reads were hard‐trimmed by 5 bases at the 3′ end. Successively, reads at least 20 bases long, and with an overall average phred quality score greater than 10 were aligned to the reference genome and transcriptome of *Mus musculus* (FASTA and GTF files, respectively, downloaded from GRCm38, Release 91) with STAR v2.5.1 (Dobin et al., 2013) with default settings for single end reads. Distribution of the reads across genomic isoform expression was quantified using the R package GenomicRanges (Lawrence et al., 2013) from Bioconductor Version 3.0.

### 4.2 Differential expression

Differentially expressed genes and proteins were identified using the R package edgeR (Robinson, McCarthy, & Smyth, 2010) from Bioconductor Version 3.0, using a generalised linear model (glm) and Quasi‐likelihood (QL) *F*‐test coupled with a Trimmed Means of M‐values (TMM) normalisation. In a differential expression analysis, a gene is marked as DE if it possesses the following characteristics: (a) at least 10 counts in at least half of the samples in one group (*above background noise* criterion, genes not meeting this criterion are marked as *absent*); (b) *p* ≤ .05; (c) log2 (fold change) ≥ 0.5.

### 4.3 Transcriptomics–proteomics integration

The integration of the transcriptomics and the proteomics data was performed using the DIABLO framework (Singh et al., 2019) from the CRAN package mixOmics (Rohart et al., 2017). Briefly, three components are chosen based on an initial fitting (function mixOmics::block.splsda) and on a 5‐Mfold, 30 repeats evaluation (function mixOmics::perf). Successively, a list of subset variables was run through a 5‐Mfold, 30 repeats tuning step (function mixOmics::tune.block.splsda) using centroids distance to select the optimal subset of variables for the final model.

## 5 EXPERIMENTAL PROCEDURES

### 5.1 Mice

Animal experiments were approved by the Kantonales Verterinäramt Zürich, Switzerland under the licence number 193/2016 and performed in accordance with ethical and legal requirements. C57BL/6 mice were kept under specific and opportunistic pathogen‐free (SPF) conditions in individually ventilated cages either in the Rodent Center HCI (RCHCI, “SR”) or in the ETH Phenomics Center (EPIC, “SE”) (ETH Zurich, Switzerland). Germ free C57BL/6 mice were kept in isolators at ETH EPIC (“GF”). Mice derived from one facility were cohoused littermates, male and 8–12 weeks old at the time of experimentation.

### 5.2 Murine organoid culture establishment and maintenance

For organoid establishment, mice were euthanized and the small intestine was isolated. Fat and vessels were removed. A ~5 cm piece of the distal jejunum was collected, washed three times in 1 mL Phosphate Buffered Saline (PBS)/0.01% Bovine Serum Albumin (BSA) and a ~2x2mm piece snap frozen in liquid nitrogen for later analysis of whole tissue (“Tissue”). The remaining tissue was opened longitudinally and washed in PBS to remove content and mucus. Subsequently, the tissue was minced and washed thoroughly in ice‐cold PBS, followed by 15 min incubation in Gentle Dissociation Reagent (Stemcell Technologies) on a rocking table at room‐temperature. Intestinal crypts were sequentially extracted by four rounds of mechanical shearing in PBS/0.01% BSA. Typically, the first and second fraction contained differentiated epithelial cells, whereas the third and fourth fraction were enriched for stem cell‐containing crypts. After filtration through a 70 μm of cell strainer, parts of fractions 1–4 were pooled, washed with PBS/0.01% BSA, pelleted (300 g, 5 min, 4°C) and half of the sample was snap frozen in liquid nitrogen for analysis of primary epithelial cells (“Epithelium”). The remaining half of fractions three and four were embedded into 50 μL Matrigel (Chemie Brunschwig) domes and kept in complete Intesticult medium (Stemcell Technologies) at 37°C, 5% CO_2_. After 3–4 days of culturing, the best‐looking wells were selected for propagation. Complete Intesticult medium was replaced every 3–4 days. Organoids were subcultured every 5–7 days by mechanical shearing in Gentle Dissociation Reagent and re‐embedding in 50 μL Matrigel domes at a 1:2 to 1:4 splitting ratio. Stable organoid cultures were cryopreserved at passage (P) 2–4 in complete medium supplemented with 10% Dimethyl Sulfoxide (DMSO). For experimentation, cryopreserved organoids were thawed, maintained in culture as above for at least 2 weeks and used for experimentation at P5‐8.

### 5.3 Cell line culture and maintenance

Mouse Embryonic Fibroblasts (MEFs) were maintained in DMEM‐Glutamax supplemented with 10% heat‐inactivated Fetal Calf Serum (FCS) and 0.05 mg/mL Streptomycin at 37°C, 5% CO_2_. m‐IC_c12_ cells were cultured in DMEM/F12 supplemented with 5 μg/mL human Insulin, 50 nM Dexamethasone, 60 nM Sodium selenite, 5 μg/mL Bovine Apo‐Transferrin, 1 nM Triiodthyronin, 40 μg/mL EGF, 20 μM HEPES and 2.5% heat‐inactivated FCS at 37°C, 5% CO_2_.

### 5.4 TNF treatment and harvesting of organoid and cell line samples

For TNF treatment, the medium was removed and replaced by the respective culture medium supplemented with 5 ng/mL murine TNF (Preprotech). For untreated controls, the medium was exchanged. After 8 hr, the medium was removed. Organoids (P5‐8) were incubated with Gentle Dissociation Reagent for 1 min to dissolve the Matrigel, but not the organoids. After pipetting up and down 10 times, extracted organoids were transferred to Eppendorf tubes (pre‐coated over night with PBS/0.01% BSA), washed in cold PBS/0.01% BSA and spun down (5 min at 300 g, 4°C). The supernatant was discarded and the pellets snap frozen in liquid nitrogen for later analysis (“Organoids”). MEFs and m‐IC_c12_ cells were washed with pre‐warmed PBS and incubated for 3 (MEFs) or 12 (m‐IC_c12_ cells) min with gentle dissociation reagent at 37°C. Subsequently, cells were detached from the bottom of the flask with the help of a cell scraper, pelleted by centrifugation (300 g, 5 min, 4°C) and the supernatant was removed. Pellets were washed with PBS/0.01% BSA, transferred to Eppendorf tubes (pre‐coated with PBS/0.01% BSA), spun down (300 g, 5 min, 4°C) and subsequently snap frozen in liquid nitrogen for later analysis.

### 5.5 RNA sequencing

Snap frozen cell/organoid pellets were lysed with the QIAShredder columns (Qiagen) according to manufacturer's instructions. RNA was isolated with the Qiagen RNeasy Micro Kit including a DNA digestion step. Subsequently, RNA concentration was assessed on a Qubit Fluorometer (Invitrogen) using a Qubit™ RNA HS Assay Kit and samples were loaded on a Fragment Analyzer (Advanced Analytical, DNF‐471‐0500 Standard Sensitivity RNA Analysis Kit [15 nt]) to determine RNA quality. RNA samples were diluted to 20 ng/mL for RNA sequencing and frozen at −80°C. Samples were processed with the TruSeq RNA sample Prep Kit v2 (Illumina, Inc., California). RNA samples (100–1,000 ng) were polyA‐enriched, reverse‐transcribed into double‐stranded cDNA and fragmented followed by end‐repair, polyadenylation and ligation of TruSeq adapters containing multiplex‐indices. Subsequently, fragments containing TruSeq adapters were selectively enriched by PCR and quality and quantity of the enriched libraries were analysed using a Qubit 1.0 fluorometer and the Calliper GX LabChip® GX (Calliper Life Sciences, Inc.). The libraries were normalised to 10 nM in Tris‐Cl 10 mM, pH 8.5 with 0.1% Tween 20. Cluster generation was performed with TruSeq PR Cluster Kit (Illumina, Inc., California) using 10 pM of the pooled normalised libraries on the cBOT. Sequencing was performed using the TruSeq SBS Kit HS4000 (Illumina, Inc., California) on an Illumina HiSeq 4,000 single end 125 bp. The transcriptome data set was uploaded to the GEO data base (Accession number GSE140703).

### 5.6 Proteome analysis

All the biological samples were suspended in 10 M Urea with complete protease inhibitor cocktail (Roche) and ultrasonically lysed in a VialTweeter device (Hielscher‐Ultrasound Technology), as previously described (Collins et al., 2017; Liu et al., 2019). The mixtures were centrifuged at 21,000 g for 1 hr and the supernatant protein amount was quantified by Bio‐Rad protein assay. Protein samples were reduced by 10 mM Tris‐(2‐carboxyethyl)‐phosphine (TCEP) for 1 hr at 37°C and 20 mM iodoacetamide (IAA) in the dark for 45 min at room temperature. All the samples were further diluted by 1:6 (v/v) with 100 mM NH4HCO3 and were digested with sequencing‐grade porcine trypsin (Promega) at a protease/protein ratio of 1:25 overnight at 37°C. The amount of the purified peptides was determined using Nanodrop ND‐1000 (Thermo Scientific) and 1.5 μg peptides were injected per a LC–MS run. The peptide samples were stored in −80°C before measurement.

An SCIEX 5600 TripleTOF mass spectrometer was interfaced with an Eksigent NanoLC. Peptides were directly injected onto a 20 cm PicoFrit emitter (New Objective, self‐packed to 20 cm), and then separated using a 90 min gradient at a flow rate of 300 nL/min (Gillet et al., 2012; Liu et al., 2019). For shotgun sequencing mode, MS1 spectra were collected in the range 360–1,460 m/z with 250 ms per scan. The 20 most intense precursors triggered MS2 spectra were collected (50–2,000 m/z for 100 ms). For SWATH mode, 64‐variable window schema was used (Collins et al., 2017; Liu et al., 2019; Ludwig et al., 2018). A dwell time of 50 ms was used for all MS2 scans after a survey MS1 scan of 250 ms, resulting in a duty cycle of ~3.45 s.

OpenSWATH (Röst et al., 2014) was used to identify peptides from all SWATH maps with statistical control at 1% FDR and then to align between SWATH maps using a novel TRIC with requantification option enabled (TRansfer of Identification Confidence; Röst et al., 2016). Because we had nine samples for tissue and epithelium type of samples analysed in this dataset (18 samples for organoid type), to further increase the protein confidence, only those peptide signals identified in more than eight samples were accepted for protein identification and quantification. To quantify the protein abundance levels across samples, we used the Top3 method (Grossmann et al., 2010; Liu et al., 2015; Ludwig, Claassen, Schmidt, & Aebersold, 2012; Williams et al., 2016). The quantitative protein matrix was rounded to the full integer value for further analysis. The input for the downstream analysis of the protein expression data was the matrix obtained by applying TMM‐normalisation to the raw count matrix. The proteome data set was uploaded to the PRIDE database (project ID PXD016339).

## CONFLICT OF INTEREST

The authors declare no potential conflict of interest.

## AUTHOR CONTRIBUTIONS

A.H. and M.E.S. prepared samples. Y.L. performed proteomic experiments. G.R., J.G. and A.H. performed data analysis. M.Z. and G.S. provided technical support. A.H., Y.L., R.A., M.E.S. and W.D.H. designed experiments. A.H., M.E.S. and W.D.H. designed the study and wrote the manuscript.

## Supporting information


**Figure S1** Sample workflow of primary samples. Tissue: distal jejunum; contains epithelium, lamina propria and submucosa. Epithelium: epithelial cell‐enriched fraction.

**Figure S2** Organoid samples cluster among each other, independent of donor microbiota. (a) Unsupervised hierarchical clustering analysis of the top 100 hits, as ranked by variance across all the samples, of the proteome data set including tissue (Tissue_I‐III, star symbol), epithelial cell‐enriched fraction (Epithelium_I‐III, circle symbol), organoid (Organoid_I‐III, hexagon symbol) samples from mice raised in SPF facility 1 (_SR, dark green), SPF facility 2 (_SE, light green) or the germ‐free facility (_GF, yellow), as well as MEFs (MEF_I‐III, blue triangle symbol) and m‐IC_c12_ cell (m‐IC_c12__I‐III, red square symbol) samples. Correlation matrix depicts Pearson correlation values between indicated samples. (b) Principal component analysis of the proteome data set as described in (a).

**Figure S3** TNF‐induced target expression in organoids. (a) Volcano plot showing TNF‐induced protein changes in the organoid sample group. (b) Volcano plot showing TNF‐induced transcript changes in the organoid sample group. Red dots: significant. Differential expression cut‐offs: log2 ratio <−0.5 or >0.5 and *p*‐value <.05.

**Figure S4** Stochastic variation overshadows low dose TNF‐induced expression changes in organoids. (a) Unsupervised hierarchical clustering analysis of the top 100 hits, as ranked by variance across all the samples, of the proteome data set including untreated (closed symbols) and TNF‐treated (5 ng/mL, 8 hr; open symbols; _T) organoid (Organoid_I‐III, hexagon symbol) samples from mice raised in SPF facility 1 (_SR, dark green), SPF facility 2 (_SE, light green) or the germ‐free facility (_GF, yellow), as well as MEFs (MEF_I‐III, blue triangle symbol) and m‐IC_c12_ cell (m‐IC_c12__I‐III, red square symbol) samples. Correlation matrix depicts Pearson correlation values between indicated samples. (b) Principal component analysis of the proteome data set as described in (a). (c) Unsupervised hierarchical clustering analysis of the top 100 hits, as ranked by variance across all the samples, of the transcriptome data set for the samples described in (a). (d) Principal component analysis of the proteome data set as described in (c).

**Figure S5** Integration of proteome and transcriptome data sets. (a) Principal component analysis of the proteome data for the three cultured sample groups of organoids (Organoid_I‐III, hexagon symbol) from mice raised in SPF facility 1 (_SR, dark green), SPF facility 2 (_SE, light green) or the germ‐free facility (_GF, yellow), MEFs (MEF_I‐III, blue triangle symbol) and m‐IC_c12_ cells (m‐IC_c12__I‐III, red square symbol). (b) Principal component analysis of the sample set as described in (a). (c) Correlation plot of log2 ratio of transcript fold changes between the Organoid sample group and m‐IC_c12_ and MEF cells (*y*‐axis) and the log2 ratio of protein fold changes between the Organoid sample group and m‐IC_c12_ and MEF cells (*x*‐axis). (d) Circle plot indicating the hits identified in the proteome–transcriptome integration as variables of PC1 and PC2 described in Figure 4 and Table S3.

**Figure S6** Organoid identifier proteins exhibit a similar expression pattern in freshly harvested primary epithelial cells and organoids. Unsupervised hierarchical clustering analysis and expression heat map of identifier proteins described in Figure 4 for organoid (Organoid_I‐III, hexagon symbol), epithelial cell‐enriched fraction (Epithelium_I‐III, circle symbol) and tissue samples (Tissue_I‐III, star symbol) from mice raised in SPF facility 1 (_SR, dark green), SPF facility 2 (_SE, light green) or the germ‐free facility (_GF, yellow), as well as MEFs (MEF_I‐III, blue triangle symbol) and m‐IC_c12_ cell (m‐IC_c12__I‐III, red square symbol) samples.

**Table S1** Significant hits of proteome analysis of TNF‐treated organoids compared to untreated organoids. Differential expression cut‐offs: log2 ratio <−0.5 or >0.5 and *p*‐value <.05.

**Table S2** Significant hits of transcriptome analysis of TNF‐treated organoids compared to untreated organoids. Differential expression cut‐offs: log2 ratio <−0.5 or >0.5 and *p*‐value <.05.

**Table S3** List of hits for Component 1 and 2 shown in Figure 4 and S5.
